# Natural Variation in Immune Responses to Neonatal *Mycobacterium bovis Bacillus* Calmette-Guerin (BCG) Vaccination in a Cohort of Gambian Infants

**DOI:** 10.1371/journal.pone.0003485

**Published:** 2008-10-22

**Authors:** Chris Finan, Martin O. C. Ota, Arnaud Marchant, Melanie J. Newport

**Affiliations:** 1 Department of Medicine, Brighton and Sussex Medical School, Falmer, Sussex, United Kingdom; 2 MRC Laboratories, Fajara, The Gambia; 3 Institute for Medical Immunology, Université Libre de Bruxelles, Charleroi, Belgium; University of Stellenbosch, South Africa

## Abstract

**Background:**

There is a need for new vaccines for tuberculosis (TB) that protect against adult pulmonary disease in regions where BCG is not effective. However, BCG could remain integral to TB control programmes because neonatal BCG protects against disseminated forms of childhood TB and many new vaccines rely on BCG to prime immunity or are recombinant strains of BCG. Interferon-gamma (IFN-γ) is required for immunity to mycobacteria and used as a marker of immunity when new vaccines are tested. Although BCG is widely given to neonates IFN-γ responses to BCG in this age group are poorly described. Characterisation of IFN-γ responses to BCG is required for interpretation of vaccine immunogenicity study data where BCG is part of the vaccination strategy.

**Methodology/Principal Findings:**

236 healthy Gambian babies were vaccinated with *M. bovis* BCG at birth. IFN-γ, interleukin (IL)-5 and IL-13 responses to purified protein derivative (PPD), killed *Mycobacterium tuberculosis* (KMTB), *M. tuberculosis* short term culture filtrate (STCF) and *M. bovis* BCG antigen 85 complex (Ag85) were measured in a whole blood assay two months after vaccination. Cytokine responses varied up to 10 log-fold within this population. The majority of infants (89–98% depending on the antigen) made IFN-γ responses and there was significant correlation between IFN-γ responses to the different mycobacterial antigens (Spearman's coefficient ranged from 0.340 to 0.675, p = 10^−6^–10^−22^). IL-13 and IL-5 responses were generally low and there were more non-responders (33–75%) for these cytokines. Nonetheless, significant correlations were observed for IL-13 and IL-5 responses to different mycobacterial antigens

**Conclusions/Significance:**

Cytokine responses to mycobacterial antigens in BCG-vaccinated infants are heterogeneous and there is significant inter-individual variation. Further studies in large populations of infants are required to identify the factors that determine variation in IFN-γ responses.

## Introduction

Tuberculosis (TB) is responsible for the deaths of over 2 million people annually [1], and efforts to control disease are failing in many high burden countries. Two main strategies underpin TB control: prevention of disease through vaccination and prevention of transmission through rapid diagnosis and treatment of infectious smear-positive pulmonary cases. *Mycobacterium bovis* bacillus Calmette-Guerin (BCG) is currently the only available vaccine against TB. It is widely given to infants as part of the WHO Expanded Programme on Immunization (EPI) in countries where TB is endemic. There is good evidence that BCG protects against disseminated forms of TB in childhood, including TB meningitis and miliary TB [2], [3]. However, the ability of BCG to prevent pulmonary TB in adults, who bear the brunt of the global burden, varies considerably and protection is lowest in countries where TB is most prevalent [4]. The Global Plan to Stop TB 2006–2015 has therefore identified research and development for new vaccines as a critical component of its strategy to eradicate TB [5].

A number of new TB vaccines are at various stages of development (reviewed by Fletcher and McShane [6]). Given the protection afforded to young children, there are strong logistical and ethical arguments to continue to use BCG in EPI vaccination programmes. Of particular interest are recombinant BCG vaccines which aim to enhance the immunogenicity of current BCG strains through the reintroduction of genes that were deleted during the attenuation of a virulent *M. bovis* strain [7]. Examples include the genes encoding the 30-kDa major secretory protein and ESAT-6 within the RD1 region of *M. bovis* [8], [9]. Alternatively, current BCG vaccines can be used to prime immune responses that are subsequently boosted with new subunit vaccines derived from immunodominant *M. tuberculosis* antigens such as antigen 85 [10], [11]. Regardless of which approach eventually proves to give the best protection for TB, robust tools for evaluating their ability to induce protective immunity are also required.

Studies in both animals and humans have demonstrated that interferon-γ (IFN-γ) is critical (though not sufficient) for immunity to *M. tuberculosis* [12], [13], [14]. For this reason, IFN-γ responses to relevant antigens are widely used as the best available correlates of protective immunity in the evaluation of new vaccines for TB [15]. IFN-γ responses also underpin new diagnostic tests for TB including the QuantiFERON Gold (Cellestis, Victoria, Australia) and T-SPOT.TB (Immunotec, Oxford, UK) assays [16] which are widely applied for the detection of latent infection. Thus, an understanding of the determinants of variation in IFN-γ responses to mycobacterial antigens is critical for the accurate interpretation of data derived from trials of both new vaccines and diagnostic tools.

It is recognised that multiple factors influence IFN-γ responses to mycobacterial infection. These include host nutritional status [17], unspecified host genetic factors [18], [19], exposure to other mycobacteria [20] and for BCG specifically, the strain and the route of immunization which can lead to variation in the immune response in infants [21]. Despite the fact that BCG has been administered since 1927, it is only recently that IFN-γ responses to BCG have been characterised in this age group. Furthermore, although it is now recognised that newborn human infants are able to generate T helper lymphocyte type 1 (Th1) responses to neonatal BCG [22], [23], the relationship between IFN-γ and the type 2 cytokines IL-13 and IL-5 have not been explored in this age group. We therefore sought to characterise the variation in IFN-γ, IL-13 and IL-5 responses to BCG in a large cohort of healthy Gambian infants who were vaccinated with BCG at birth.

## Methods

### Study population

The study was conducted at the Medical Research Laboratories, The Gambia, with approval from The Gambia Government/Medical Research Council (MRC) Ethics Committee.

With the mother's informed consent, newborn infants were enrolled within 24 hours of birth at the Royal Victoria Teaching Hospital (the referral hospital in the capital Banjul) or at one of two health centres (Serrekunda and Fajikunda) in the same district. Consent was given verbally due to the high level of illiteracy within the population – this was approved by The Gambia Government/Medical Research Council (MRC) Ethics Committee. Twins and neonates born to mothers with systemic infection at the time of delivery, or with a history suggestive of contact with TB and those with congenital defects or with birth weight less than 2.5 kg were excluded from the study. All subjects were vaccinated at enrolment (i.e. within 24 hours of birth) with 0.05 ml of BCG (Aventis Pasteur, Lyon, France) intradermally in the left deltoid region. Other vaccines were given as per the EPI programme.

### Whole blood assay and antigens

BCG immunogenicity was evaluated 2 months after vaccination using a whole blood assay, due to the limited amount of blood available [24]. One to two ml of heparinized blood was diluted in 9 volumes of RPMI 1640 medium (BioWittaker, Verviers, Belgium) supplemented with 100 U/ml penicillin and 100 mg/ml streptomycin (LifeTechnologies, Paisley, Scotland). Diluted blood was incubated for 2 or 6 days in 96-well plates (200 µl/well) (Becton Dickinson, Rutherford, NJ) containing antigen preparations in duplicates [25]. Mycobacterial antigens included *M. tuberculosis* purified protein derivative (PPD, RT49, 10 µg/ml; Statens Serum Institut, Denmark), *M. tuberculosis* short term culture filtrate (STCF, 10 µg/ml; a gift from Dr Peter Andersen, Statens Serum Institut, Denmark), killed *M. tuberculosis* (KMTB) and *M. bovis* BCG Ag85 complex (both at 10 µg/ml, and given by Dr Kris Huygen, Institut Pasteur du Brabant, Belgium). Wells containing medium alone or phytohaemaglutinin (PHA-L, Sigma Chemicals) were used as the negative and positive controls, respectively. We chose mainly *M. tuberculosis* antigens (PPD, KMTB and STCF) because we were primarily interested in immunity induced by BCG in the context of protection against TB, and Ag85 was chosen as it is a candidate subunit vaccine antigen.

### Cytokine assays

Cytokine concentrations in supernatants collected on day 2 (PHA) or day 6 (medium and antigens) were measured by enzyme-linked immunosorbent assay, using commercially available kits (IFN-γ and IL-5; BioSource Europe, Fleurus, Belgium; IL-13; Diaclone, Besançon, France). The lower limit of detection was 10 pg/ml for IFN-γ and 1.5 pg/ml for IL-5 and IL-13. These values were used as the cut off to determine non-responders. Background production of cytokines measured in control wells was subtracted from that measured in Ag-stimulated wells and duplicates for each antigen averaged.

### Tuberculin skin testing

Tuberculin skin testing (TST) was performed after phlebotomy on the volar surface of the forearm with 2TU of PPD (Statens Serum Institute, Belgium) using the Mantoux method [26]. *Candida albicans* antigen (Evans Medical Limited, UK) was used as the skin test control antigen. The length of the maximum transverse induration at 48–72 hours was taken as the response.

The vertical and horizontal size of the BCG scar was also measured and the mean recorded at the time of cytokine assessment.

### Data analysis

Logarithm transformation was applied to all cytokine data before statistical analysis. All statistical analyses and graphics were done using the R software package (http://www.r-project.org/). The Kruskal-Wallis test was used to compare median levels of IFN-γ response to PPD for data grouped by TST response size. Spearman's rank correlation coefficient was used to measure correlation between cytokine responses to mycobacterial antigens and PHA, TST response and BCG scar size. Non-responders were excluded from correlation analysis. We arbitrarily used cut-off of ρ = 0.3–0.5 as evidence of weak correlation and ρ≥0.5 as evidence of strong correlation.

## Results

A total of 236 babies were studied of which 124 (53%) were female. Only healthy babies who were normal on examination without clinical evidence of congenital abnormalities or infection were enrolled. The average birth weight was 3.189 (±0.49) kg for male infants and 3.185 (±0.55) kg for female infants. A BCG scar was present in 221 (94%) of the babies.

### Cytokine responses

**Figure 1:**
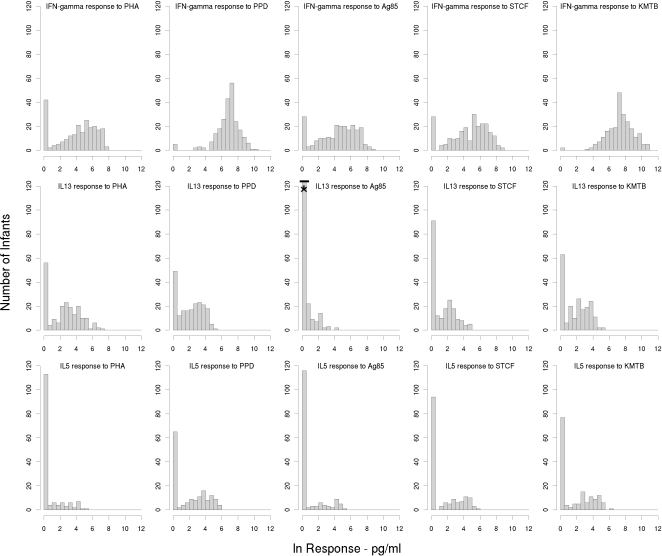
Histograms showing distribution of cytokine responses to mycobacterial antigens and PHA in BCG vaccinated infants. Table 1 indicates total numbers of infants included in each histogram plot. The asterisk denotes >120 babies did not make IL-13 responses to Ag85 (n = 138). IFN-γ: interferon-gamma; IL-13: interleukin-13; IL-5: interleukin-5; PHA: phytohaemagglutinin; PPD: purified protein derivative; Ag85: antigen 85; STCF: short term culture filtrate; KMTB: killed *Mycobacterium tuberculosis*.

To evaluate cellular immune responses to neonatal BCG we measured IFN-γ, IL-13 and IL-5 responses to the mycobacterial antigens PPD, Ag85, STCF and KMTB, and compared them to PHA responses. IFN-γ responses were measured in all 236 (100%) infants. Due to limited volumes of supernatant collected in some infants, IL-13 and IL-5 responses to the various mycobacterial antigens could only be measured in 200 (85%) and 155 (66%) babies respectively. The geometric means and 95% confidence intervals for the cytokine responses are presented in Table 1. Histograms showing the distribution of IFN-γ, IL13 and IL-5 responses to PHA, PPD, Ag85, STCF and KMTB are shown in Figure 1. There was no correlation between birth weight and magnitude of response for any of the responses we studied and there were no differences between male and female responses within the total population (data not presented).

**Table 1:** Cytokine responses to mycobacterial antigens in BCG vaccinated infants.

Cytokine	Antigen^a^	Numbers available^b^	Non-responders (%)^c^	Geometric mean (pg/ml, 95% CI)
IFN-γ	PHA	232	42 (18)	58.37 (42.59–80.02)
N = 236	PPD	236	5 (2)	845.39 (692.67–1031.79)
	Ag85	228	26 (11)	76.23 (56.14–103.52)
	STCF	236	27 (11)	99.15 (73.21–134.28)
	KMTB	236	2 (1)	1574.70 (1278.33–1939.78)
IL-13	PHA	200	55 (28)	11.79 (8.99–15.47)
N = 200	PPD	200	45 (23)	8.02 (6.46–9.95)
	Ag85	200	138 (69)	1.61 (1.42–1.82)
	STCF	200	87 (44)	3.64 (2.99–4.43)
	KMTB	200	61 (31)	6.45 (5.20–8.01)
IL-5	PHA	153	110 (72)	1.97 (1.60–2.43)
N = 155	PPD	154	64 (42)	7.83 (5.68–10.80)
	Ag85	154	115 (75)	2.18 (1.72–2.78)
	STCF	155	93 (60)	3.89 (2.90–5.21)
	KMTB	153	76 (50)	5.57 (4.08–7.61)

a Concentration 10 µg/ml.

b Discrepancies between number available and total number in study are due to technical failure for a few samples in the IFN-γ and IL-5 groups.

c non-responders defined as cytokine level of 0 or less after background subtracted; percentages rounded up to nearest integer.

IFN-γ: interferon-gamma; IL-13: interleukin-13; IL-5: interleukin-5; PHA: phytohaemagglutinin; PPD: purified protein derivative; Ag85: antigen 85; STCF: short term culture filtrate; KMTB: killed *Mycobacterium tuberculosis*.

The most striking observation is that our data show significant inter-individual variation of up to 10 log-fold differences for all three cytokine responses to both PHA and the mycobacterial antigens tested. Two response patterns emerged that could be differentiated by the numbers of non-responders, which were notably higher for IL-13 and IL-5 responses to mycobacterial antigens (Figure 1). For phenotypes where the majority of infants responded, the data approximated to a normal distribution after log transformation. IFN-γ was induced in response to mycobacterial antigens in the majority of infants. Over 98% of infants produced IFN-γ in response to PPD and KMTB and 89% of infants made IFN-γ responses to Ag85 and STCF. These data are consistent with our previous work which demonstrated that infants generated predominantly Th1 responses to mycobacterial antigens following neonatal BCG [22], [27].

In contrast to the IFN-γ responses, IL-13 and IL-5 responses were generally low. There were also considerably more non-responders in these groups (Table 1 and Figure 1). This is consistent with previously published data and again reflects the fact that BCG is a potent inducer of Th1 responses, even in young infants [23], [27]. As with IFN-γ, a higher proportion of infants responded to PPD and KMTB than to the secreted antigens STCF and Ag85. Thus, 77% and 69% of infants made an IL-13 response to PPD and KMTB respectively while only 56 and 31% responded to STCF and Ag 85 respectively. This pattern was seen also in IL-5 responses with 68% and 50% responding to PPD and KMTB respectively, but only 40% and 25% responding to STCF and Ag85 respectively. Within the group of responders, there was again a 6 log-fold range in the magnitude of response that approximated to a normal distribution when non-responders were excluded. Responses to PPD and KMTB were generally higher for all cytokines compared to STCF and Ag85, reflecting the fact that PPD and KMTB contain many more antigens that will stimulate more T cell clones.

PHA was included as a positive control for this study yet only 82%, 72% and 28% of infants produced IFN-γ, IL13 or IL-5 respectively to this antigen. However, given that over 98% of infants had IFN-γ responses to PPD and the negative controls were negative, the low PHA responses reflect a true biological response rather than any technical problem with the assay used.

### Cytokine correlations

**Figure 2:**
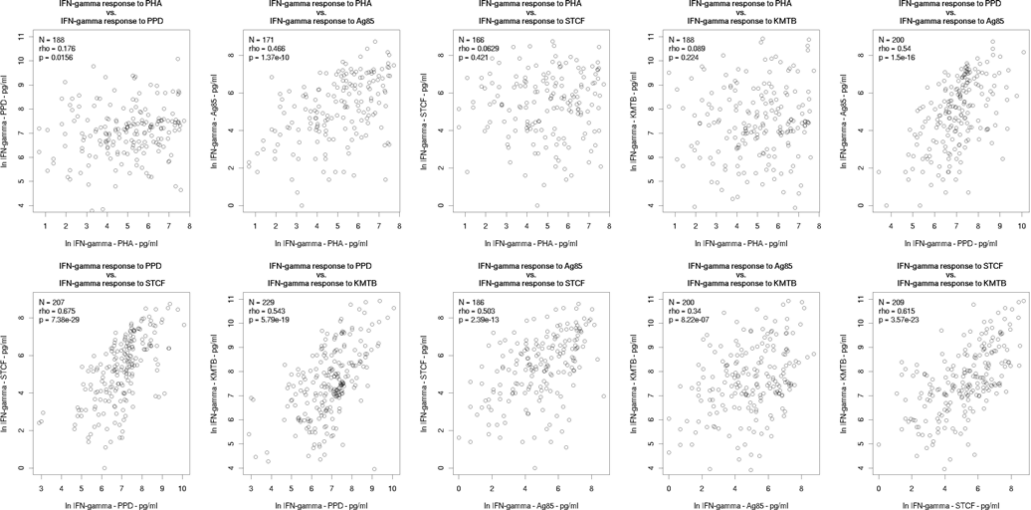
Correlations between interferon-γ responses to PHA and various mycobacterial antigens. N = number of infants who responded to both antigens considered. Rho: Spearman's rank correlation coefficient; IFN-γ: interferon-gamma; PHA: phytohaemagglutinin; PPD: purified protein derivative; Ag85: antigen 85; STCF: short term culture filtrate; KMTB: killed *Mycobacterium tuberculosis*.

**Figure 3:**
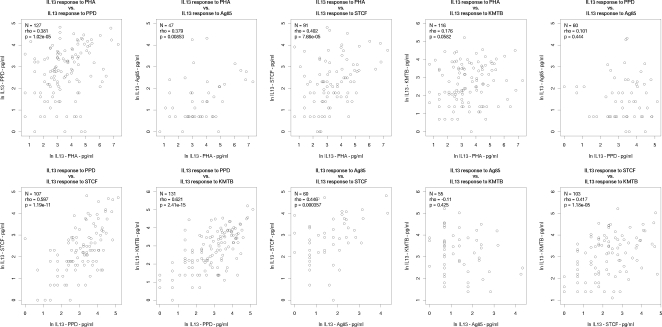
Correlations between interleukin-13 responses to PHA and various mycobacterial antigens. N = number of infants who responded to both antigens considered. Rho: Spearman's rank correlation coefficient; IL-13: interleukin-13; PHA: phytohaemagglutinin; PPD: purified protein derivative; Ag85: antigen 85; STCF: short term culture filtrate; KMTB: killed *Mycobacterium tuberculosis*.

**Figure 4:**
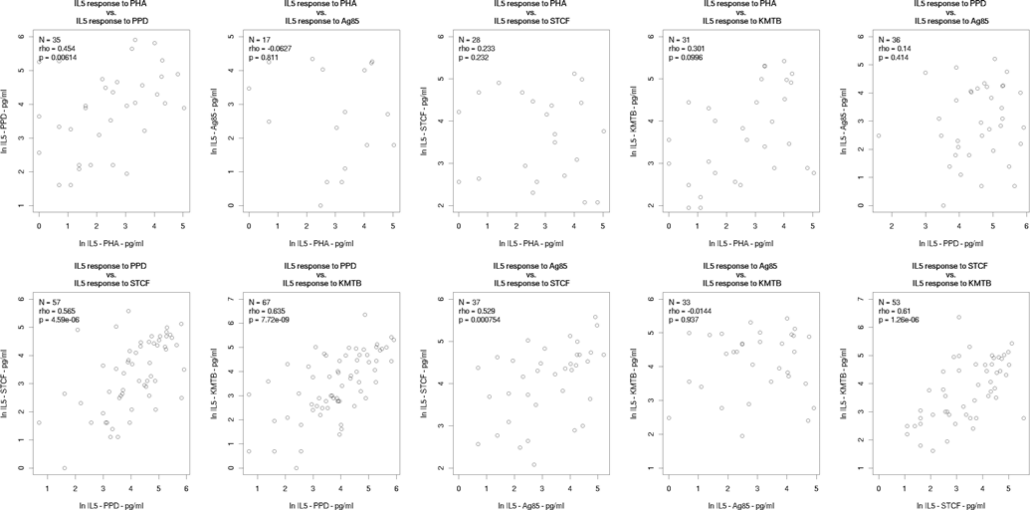
Correlations between interleukin-5 responses to PHA and various mycobacterial antigens. N = number of infants who responded to both antigens considered. Rho: Spearman's rank correlation coefficient; interleukin-5; PHA: phytohaemagglutinin; PPD: purified protein derivative; Ag85: antigen 85; STCF: short term culture filtrate; KMTB: killed *Mycobacterium tuberculosis*.

**Figure 5:**
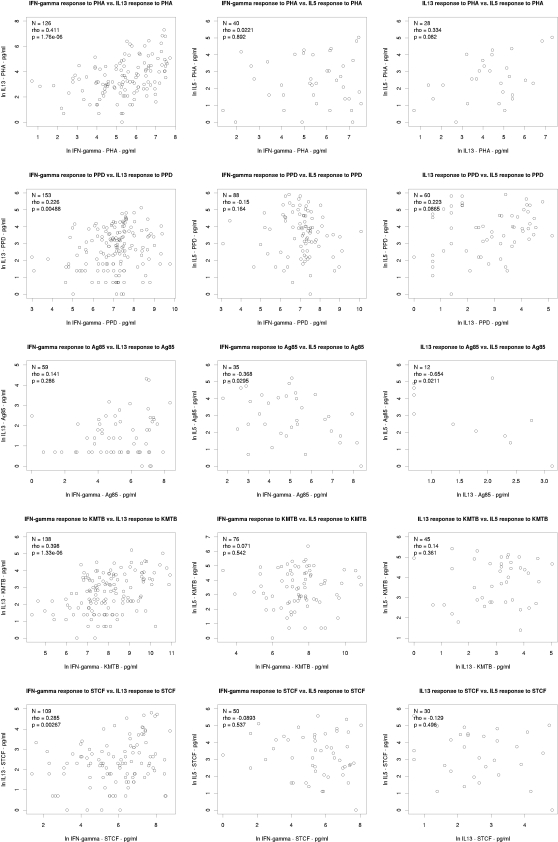
Correlations between the three cytokine responses to specific antigens. N = number of infants who responded to both cytokines considered. Rho: Spearman's rank correlation coefficient; IFN-γ: interferon-gamma; IL-13: interleukin-13; IL-5: interleukin-5; PHA: phytohaemagglutinin; PPD: purified protein derivative; Ag85: antigen 85; STCF: short term culture filtrate; KMTB: killed *Mycobacterium tuberculosis*.

Next, we examined the relationship between cytokine responses to the various antigens tested. Non-responders were excluded from the analysis to avoid non-responder correlations artificially skewing the correlation coefficients. We firstly tested for correlation between the magnitude of cytokine response to the various antigens for each cytokine separately. Figures 2, 3 and 4 show scatter plots for each pairwise correlation for IFN-γ, IL-13 and IL-5 respectively and the correlation results are summarised in Table 2. We then considered correlation between the three cytokines for each antigen separately (Table 3 and figure 5).

**Table 2:** Correlations for individual cytokine responses to various mycobacterial antigens and PHA in BCG vaccinated infants.

	*IFN-γ PHA*			*IFN-γ PPD*			*IFN-γ Ag85*			*IFN-γ STCF*			*IFN-γ KMTB*		
	N^a^	ρ^b^	p value^c^	N	ρ^b^	p value	N	ρ^b^	p value	N	ρ^b^	p value	N	ρ^b^	p value
**IFN-γ PHA**	-	-	-	188	0.176	0.0156	171	**0.466**	**1.37×10^−10^**	166	0.0629	0.421	188	0.089	0.224
**IFN-γ PPD**				-	-	-	200	**0.540**	**1.5×10^−16^**	207	**0.675**	**7.38×10^−29^**	229	**0.543**	**5.79×10^−19^**
**IFN-γ Ag85**							-	-	-	186	**0.503**	**2.39×10^−13^**	200	**0.340**	**8.22×10^−7^**
**IFN-γ STCF**										-	-	-	209	**0.615**	**3.57×10^−23^**
**IFN-γ KMTB**													-	-	-

	*IL-13 PHA*			*IL-13 PPD*			*IL-13 Ag85*			*IL-13 STCF*			*IL-13 KMTB*		
	N^a^	ρ^b^	p value	N^a^	ρ^b^	p value	N^a^	ρ^b^	p value	N^a^	ρ^b^	p value	N^a^	ρ^b^	p value
**IL-13 PHA**	-	-	-	127	**0.381**	**1.02×10^−5^**	47	**0.379**	**0.009**	91	**0.402**	**7.66×10^−5^**	116	0.1876	0.0582
**IL-13 PPD**				-	-	-	60	0.101	0.444	107	**0.597**	**1.19×10^−11^**	131	**0.621**	**2.41×10^−15^**
**IL-13 Ag85**							-	-	-	60	**0.446**	**0.3.57×10^−4^**	55	−0.11	0.425
**IL-13 STCF**										-	-	-	103	**0.417**	**1.18×10^−5^**
**IL-13 KMTB**													-	-	-

	*IL-5 PHA*			*IL-5 PPD*			*IL-5 Ag85*			*IL-5 STCF*			*IL-5 KMTB*		
	N^a^	ρ^b^	p value	N^a^	ρ^b^	p value	N^a^	ρ^b^	p value	N^a^	ρ^b^	p value	N^a^	ρ^b^	p value
**IL-5 PHA**	-	-	-	35	**0.454**	**0.006**	17	−0.0627	0.811	28	0.233	0.232	31	0.301	0.0996
**IL-5 PPD**				-	-	-	36	0.14	0.414	57	**0.565**	**4.59×10^−6^**	67	**0.635**	**7.72×10^−9^**
**IL-5 Ag85**							-	-	-	37	**0.529**	**7.54×10^−4^**	33	−0.0144	0.937
**IL-5 STCF**										-	-	-	53	**0.610**	**1.26×10^−6^**
**IL-5 KMTB**													-	-	-

a Number of infants for whom both cytokine data were available for correlation analysis.

b Spearman rank-order correlation coefficient.

ρ values>0.3 with corresponding p values≤0.05 are shown in bold.

IFN-γ: interferon-gamma; IL-13: interleukin-13; IL-5: interleukin-5; PHA: phytohaemagglutinin; PPD: purified protein derivative; Ag85: antigen 85; STCF: short term culture filtrate; KMTB: killed *Mycobacterium tuberculosis*.

**Table 3:** Correlations between different cytokines responses to individual mycobacterial antigens.

Antigen	cytokine	*IL-13*			*IL-5*		
		N^a^	ρ^b^	p value	N^a^	ρ^b^	p value
**PHA**	**IFN-γ**	126	**0.411**	**1.76×10^−6^**	40	0.022	0.892
	**IL-13**	-	-	-	28	0.334	0.082
**PPD**	**IFN-γ**	153	0.226	0.005	88	−0.150	0.164
	**IL-13**	-	-	-	60	0.223	0.0865
**Ag85**	**IFN-γ**	59	0.141	0.286	35	**−0.368**	**0.0295**
	**IL-13**	-	-	-	12	**−0.654**	**0.0211**
**STCF**	**IFN-γ**	109	0.285	0.0027	50	−0.0893	0.537
	**IL-13**	-	-	-	30	−0.129	0.496
**KMTB**	**IFN-γ**	138	**0.398**	**1.33×10^−6^**	76	0.071	0.542
	**IL-13**	-	-	-	45	0.14	0.361

a Number of infants for whom both cytokine data were available for correlation analysis.

b Spearman rank-order correlation coefficient.

ρ values>0.3 with corresponding p values≤0.05 are shown in bold.

IFN-γ: interferon-gamma; IL-13: interleukin-13; IL-5: interleukin-5; PHA: phytohaemagglutinin; PPD: purified protein derivative; Ag85: antigen 85; STCF: short term culture filtrate; KMTB: killed *Mycobacterium tuberculosis*.

#### Intra-cytokine correlations: PHA responses

IFN-γ responses to PHA showed correlation with Ag85 IFN-γ responses (Spearman rank correlation coefficient ρ = 0.466, p = 1.37×10^−10^) but no correlation with IFN-γ responses to the other mycobacterial antigens PPD, KMTB and STCF (Figure 2).

Interleukin-13 production in response to PHA correlated with IL-13 responses to PPD (ρ = 0.381, p = 1.2×10^−5^), Ag 85 (ρ = 0.379, p = 0.0085) and STCF (ρ = 0.402, p = 7.66×10^−5^) (Figure 3). A weaker correlation was observed with IL-13 production in response to KMTB, though this did not reach statistical significance.

There was no correlation between IL-5 responses to PHA and IL-5 responses to any of the mycobacterial antigens tested, although the numbers available to for analysis were too small in this group to draw any meaningful conclusions (Figure 4).

#### Intra-cytokine correlations: mycobacterial antigen responses

When responses to mycobacterial antigens were considered for each cytokine individually, we found strong correlation between IFN-γ responses to PPD and IFN-γ responses to Ag85, KMTB and STCF, all of which had a Spearman's coefficient of >0.5 (p<10^−15^–10^−28^) (Figure 2 and Table 2). IFN-γ responses to STCF and KMTB were also strongly correlated with ρ = 0.615 (p = 3.57×10^−23^), whilst IFN-γ induced by Ag85 correlated with both STCF (ρ = 0.503, p = 2.39×10^−13^) and KMTB (ρ = 0.340, p = 8.22×10^−7^). In summary, all IFN-γ responses to all mycobacterial antigens correlated with each other. Some correlations were stronger than others, with correlations between PPD and the other mycobacterial antigens being the strongest.

Interleukin-13 responses (shown in figure 3) to PPD correlated strongly with IL-13 responses to STCF and KMTB, but not to Ag 85. However, 69% of infants did not make IL-13 responses to Ag 85, which may also explain the lack of correlation between IL-13 responses to KMTB and Ag85. We did observe correlation between IL-13 responses to STCF and Ag 85 (ρ = 0.446, p = 3.6×10^−4^) and IL-13 responses to KMTB and STCF (ρ = 0.417, p = 1.18×10^−5^).

Given the generally low response rates for IL-5 and the smaller number of samples tested, it is perhaps not surprising that we did not observe strong correlations for IL-5 compared to IFN-γ and IL-13 (figure 4). Even so, IL-5 responses to PPD were strongly correlated with IL-5 responses to both STCF (ρ = 0.565, p = 4.59×10^−6^) and KMTB (ρ = 0.635, p = 7.72×10^−9^),but not Ag 85, and likewise there was strong correlation between IL-5 responses to STCF and KMTB (ρ = 0.610, p = 1.26×10^−6^) (Table 2).

#### Inter-cytokine correlations

Next we analysed correlations for the different cytokines to individual antigens. The results are presented in figure 5 and summarised in table 3. It should be noted that for some combinations the numbers of individuals who had made more than one cytokine response to a given antigen were small, and results should be interpreted with caution. Thus, IL-13 and IL-5 responses to Ag85 were apparently strongly inversely correlated (ρ = −0.654 p = 0.0211) but only 12 subjects (5%) were included in the analysis. Otherwise, we did not observe any strong correlation (i.e. ρ≥0.5) between any of the cytokine responses to individual antigens. IFN-γ and IL-13 responses to both PHA and KMTB were weakly correlated and the same responses for PPD and STCF were just below our arbitrary cut off for reporting weak correlation at ρ = 0.226, p = 0.005 and ρ = 0.285, p = 0.003 respectively. Likewise, there was a weak correlation between IL-13 and IL-5 responses to PHA (ρ = 0.334) but this was not statistically significant.

### Cytokine responses, tuberculin skin tests and BCG scar

**Figure 6:**
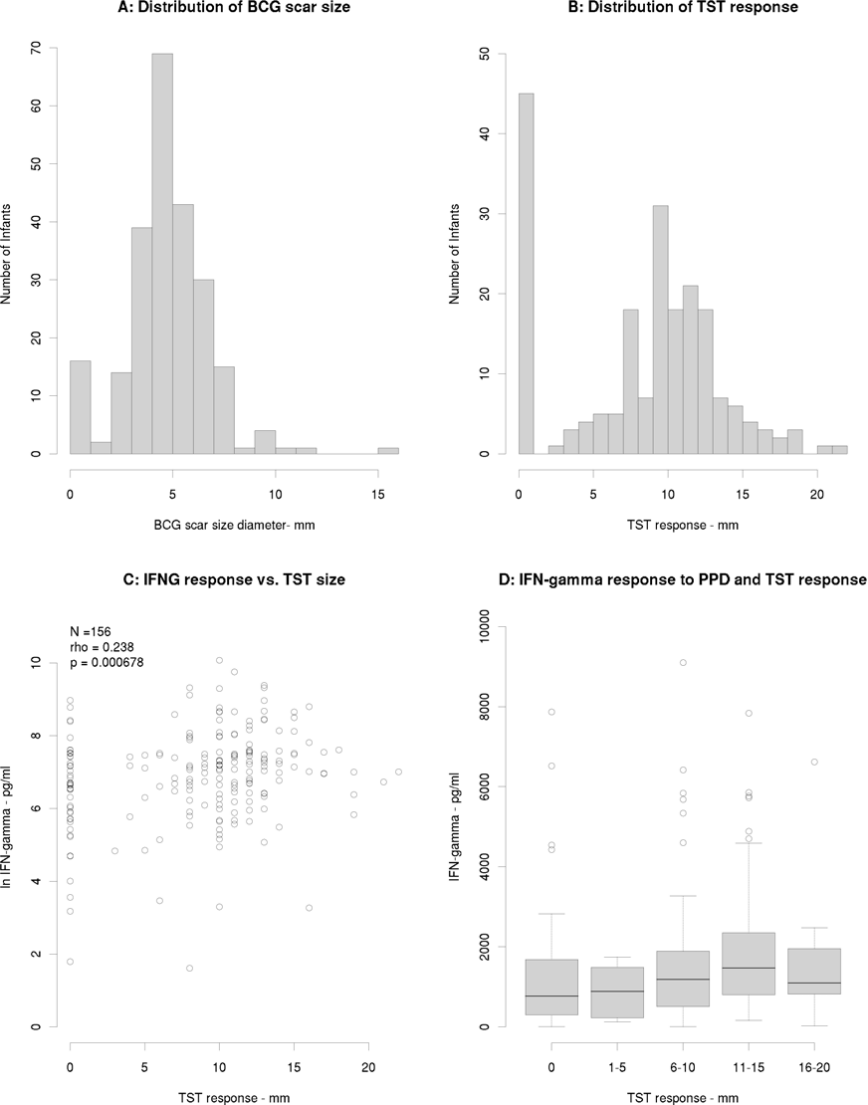
A) Histogram showing distribution of BCG scar size in 236 infants measured at 2 months of age following BCG at birth. B) Histogram showing distribution of average TST size in 202 infants tested at 2 months of age following BCG at birth. C) Scatterplot showing correlation between interferon-γ and TST responses to PPD. D) Box and whisker plot showing median IFN-γ concentrations (pg/ml) in supernatant following stimulation with PPD in infants grouped according the size of TST response. IFN-γ: interferon-gamma; PPD: purified protein derivative; TST: tuberculin skin test.

The average diameter of the BCG scar was recorded in the 236 babies in whom cytokine studies were performed (Figure 6A). The mean BCG scar size was 5.19 (±2.18) mm. There was no correlation between cytokine response and size of BCG scar (data not shown). Tuberculin skin tests (TST) were measured in 202 babies at 2 months of age - 34 (14.4%) of the 236 babies who underwent skin testing at the time of blood sampling were not available 48–72 hours later for skin test reading. The mean response was 8.11±5 mm. Forty-three babies (21.2%) did not react to intradermal PPD and 98 (49%) babies had a response of ≥10 mm (Figure 6B). There was no correlation between TST diameter and magnitude of IFN-γ response to PPD in the 202 babies for whom both data were available (ρ = 0.238, p = 6.78×10^−4^; Figure 6C). We also compared the median IFN-γ concentration between groups when infants were grouped according to the conventional cut-offs for interpreting TST responses: 0, <5 mm, 6–10 mm, 11–15 mm and 16–20 mm (Figure 6D). Again, we found no association between magnitude of in vitro IFN-γ and TST response (Figure 6D). All 43 babies who did not respond to intradermal PPD responded to mycobacterial antigens *in vitro* (Figure 6C).

## Discussion

Although BCG has been administered to millions of infants around the world, the immune responses induced by BCG in this age group are poorly characterised compared to other vaccines. Given the drive for new TB vaccine strategies that are likely to include BCG, whether as a recombinant BCG strain or in its current form with a boosting vaccine such as the MVA85, there is a need to understand immunity to BCG in greater depth in order to fully evaluate newer vaccines. Our previous work in The Gambia has shown that responses to BCG given at birth were predominantly T helper lymphocyte type 1 responses and of a similar magnitude to responses induced in adults [22], [27]. This has subsequently been confirmed by other groups in other settings and using different methodology [23], [28]. These findings have now been replicated in a much larger cohort presented in this paper where we have demonstrated that the majority of infants (89–98% of infants depending on the antigen) developed antigen specific IFN-γ response to mycobacterial antigens following BCG vaccination. We have also reported data on IL-13 and IL-5 responses which have not been described in previous studies. We found a large number of infants did generate IL-13 and IL-5 response to both PHA and mycobacterial antigens. Given the high IFN-γ response rate to PPD in the same infants, we are confident that this does not reflect a technical problem with the assay. The fact that there are more non-responders for IL-13 and IL-5 is to be expected because we are studying a Th1 response and this has been reported by us and others previously [22], [23]. The higher frequency of non-responders for some antigen/cytokine combinations may also relate to the fact we used a whole blood assay – using purified peripheral blood mononuclear cell assays would have increased sensitivity by increasing the numbers of responsive cells being stimulated, but was not practical for the scale of study we wished to conduct when blood volumes were limited due to the young age of the study participants. Widespread surveys of PHA responses in this age group have not previously been reported. Given our PHA data, we would suggest that PHA is not the best positive control antigen for this age group and alternative antigens such as Stapylococcus aureus Cowan should be investigated.

We have also assessed TST reactivity post-BCG in a large infant sample and found that 78% of babies had a positive TST 2 months after BCG. This is similar to other Sub-Saharan African populations [29]. Since exposure to non-tuberculous mycobacteria is likely to be low in this population in the first 2 months of life (see below for further discussion of this) this probably reflects recent BCG vaccination. However, our previous work has shown that by the age of 5 months, approximately 20% of unvaccinated infants have developed a positive TST indicating that exposure to NTM may also contribute [30]. Further longitudinal studies of TST responses following BCG in TB-endemic regions are warranted. In this work we have expanded our previous studies in The Gambia and demonstrated that there was no correlation between TST response and the magnitude of *in vitro* IFN-γ responses to PPD in a much larger sample [30]. All 43 infants who did not respond to TST in this study responded to PPD *in vitro*. This is consistent with previous studies in this Gambian population [30], though others have found good correlation between 6-day IFN-γ responses *in vitro* and Mantoux induration in healthy Malawian adults [31]. These differences may be explained by differences in the genetic background of the two populations that were studied or the age of the study subjects. Adults living in countries such as Malawi have been exposed chronically to mycobacterial antigens since childhood. Chronic antigen exposure probably increases TST and in vitro IFN-γ responses in parallel giving rise to the positive correlation observed by Black et al [31]. Meanwhile, in an infant population where NTM exposure is much lower, the dissociation between TST and in-vitro responses suggests that different mechanisms are involved in the two types of response. Both assays rely on the production of cytokines by antigen specific T cells but the TST is a delayed hypersensitivity response that involves injection of antigen into the dermis triggering the infiltration of specific T lymphocytes into the region in a positive response. There may be factors that influence this response – e.g. the local chemokine milieu, that are not relevant to in vitro responses using blood.

The large number of infants enrolled in this study has revealed two important findings that have not been described previously in this age group. Firstly, the new data presented in this paper highlight the extent of inter-individual variation there is in the immune response to BCG in infants, the main recipients of BCG vaccination. Secondly, we have identified a number of correlations between responses to different antigens that are worthy of further discussion and investigation.

A number of factors, both environmental and host-related, influence immune responses to vaccination. These include the nutritional status of the vaccinee, exposure to other antigens through intercurrent infections or administration of other vaccines, chronic immunosuppressive diseases such as HIV/AIDS, immunsupressive drug therapy and host genetic factors. Ideally, the design of a study such as ours should take all these factors into account and ensure appropriate control measures are included. However, there are many uncertainties – there will be influences that have yet to be defined, and for those that are defined we do not know how they interact with each other to influence the immune responses. For these reasons, human studies will inevitably have limitations, but we will focus here on issues of particular relevance to the study of immune responses to BCG in an infant cohort.

It is well documented that exposure to other mycobacteria species, including *M. tuberculosis*, can influence immune responses to BCG [32]. We believe that *M. bovis* BCG was the first mycobacterium the infants in this study were exposed to and the immune responses we measured were primary responses: we have previously found that cord blood samples stimulated with mycobacterial antigens were unresponsive to when evaluated prior to a related previous study [22]. Furthermore, 2 and 4 month old Gambian infants who had not received BCG vaccination did not respond to mycobacterial antigens using similar assays [22], [33]. The babies in this study were exclusively breastfed for the first two months of life and had limited contact with other environmental sources of non-tuberculous mycobacteria. In terms of exposure to *M. tuberculosis*, we excluded infants where there was a family history of TB or where the mother or any other household member has symptoms suggestive of TB. The prevalence of TB is also relatively low in this community, compared to other sub-Saharan African countries [34]. For these reasons we think it unlikely that the immune responses we measured were appreciably influenced by infection with other mycobacteria species.

The HIV prevalence in the antenatal clinic where these infants were born was very low (<1%) [35], excluding HIV infection as a major influence on vaccine responses in this population. Regarding exposure to other antigens such as other EPI vaccines, the infants did receive polio and hepatitis B vaccines at birth but all babies received the same dose of the same batch of vaccine from the same manufacturer, at the same time as they received BCG. There is much interest currently in the relationship between helminth infection and response to BCG. Treatment of asymptomatic adult carriers of intestinal helminths led to increased immunogenicity of BCG compared to untreated controls in Ethiopia [36], while data from Kenya suggested that in-utero exposure to *Wuchereria bancrofti* or *Schistosoma haematobium* antigens down-regulated antigen specific IFN-γ production after neonatal BCG vaccination [37]. There are no data on helminth infections in very young infants on The Gambia but given that all infants in this study were breastfed and too young to have much soil contact it is unlikely that many study subjects acquired helminth infection in the first 2 months of life. Meanwhile, schistosomiasis is rarely encountered in the coastal region of The Gambia where this study took place (there are cases to the far East of the country who may migrate to the coast) and filariasis is not endemic in The Gambia.

Nutritional factors may also have influences responses. Nutritional status as a modifying influence on immune responses is difficult to assess accurately in this age group as well evaluated parameters have not been determined. However, we excluded low birth weight babies (birth weight<2.5 kg) from the study, all babies were born to healthy mothers, were healthy at birth and had gained weight appropriately when reviewed at 2 months of age. All babies were exclusively breastfed during the period of the study. There was no correlation between birth weight and immune response.

There is a large body of evidence that host genetic factors influence immune responses both to infectious agents and to vaccines [38], [39], [40]. We have shown previously that *in vitro* IFN-γ responses to PPD in a cohort of infant twins from the same region as the subjects included in this study are heritable suggesting that host genetic factors are major determinants of variation in IFN-γ responses [19]. Approximately 40% of the variation in IFN-γ responses to PPD following BCG were attributable to host genetic factors. Whilst we cannot quantify the relative contribution of each environmental factor mentioned above, genetic factors are likely to be the single biggest contributor the the observed variation, in this study population at least. Further studies are now required to evaluate the role of genetic factors in other populations.

Although caution is required when extrapolating finding from one population to others, it is likely that genetic factors will be important in all populations and what will vary is the relative contribution of genetic factors to the overall variation and possibly the specific genes involved. With this caveat, our data suggest that host genetic factors may be important confounders when IFN-γ responses are used as markers of immunity to new vaccines undergoing efficacy evaluation in this age group, especially if a prime-boost strategy is adopted that includes standard BCG. Low IFN-γ responses to mycobacterial antigens following BCG may not reflect poor immmunogenicity of the vaccine *per se*, rather that the host is genetically a low responder. Although IFN-γ is an important marker of immunogenetioity following BCG vaccination, the potential application of our findings to the clinical testing of new vaccines for TB will depend on whether there is any correlation *in vivo* between the magnitude of the IFN-γ response to vaccination and protection against TB, which is currently not known. Although there are clear data suggesting that complete absence of IFN-γ responses (as in children in whom the IFN-γ receptor is completed disrupted) leads to severe and disseminated mycobacterial infection [41], [42], there are no data to suggest that the level of protection directly correlates with level of IFN-γ response. If these parameters do correlate, genetic testing could identify those at risk of vaccine failure who may benefit from other measures to prevent the development of TB. Alternatively, host genotype at key genetic loci may be further parameters to consider when analysing data from vaccine trials.

Recent developments in genetics such as the Human Genome Project [43], the Single Nucleotide Polymorphism (SNP) Consortium [44] and the International HapMap project [45], [46], coupled with technological advances, have allowed large scale genotyping to be undertaken as exemplified by the Wellcome Trust Case Control Consortium [47]. It is therefore now possible to study the basis of genetic variation in IFN-γ response and to identify DNA variants that are associated with the magnitude of the response. This in turn may allow the identification of low responders in advance, in the same way that pharmacogenetics allows the identification of individuals at risk of a specific adverse drug reaction [48]. These findings may also have relevance for IFN-γ based diagnostic tests where the hope is that quantification of level of response may be helpful to distinguish latent infection from active disease.

The other new findings from this study are the correlations between the cytokine responses to various mycobacterial antigens and PHA. The antigens selected included PPD which is a mixed protein extract derived from *M. tuberculosis*; *M. bovis* BCG Ag85 complex, an immunogenic secreted antigen chosen for its current popularity as a subunit vaccine candidate and *M. tuberculosis* STCF which is rich in low molecular weight proteins secreted by dividing bacteria.

The correlations observed for cytokine responses to the various mycobacterial antigens could reflect two mechanisms. Firstly, for some, e.g. Ag85 and STCF there is overlap in the antigens stimulating the cytokine response through the same antigen specific T cell receptors, as Ag 85 is found in STCF. Thus responses to Ag85 and STCF were strongly correlated for all three cytokines investigated. Likewise, responses to PPD and KMTB, which overlap in terms of the antigens represented, were also strongly correlated for all three cytokines. Secondly, for antigens that are not related (e.g. Ag85 and KMTB), it is unlikely that responses are mediated through shared receptors and we postulate that factors that influence the cytokine response to these antigens do so in an antigen independent way. This could involve co-stimulatory molecules or molecules involved in downstream signalling pathways shared by all cells irrespective of antigen specificity, once the T cell receptor has engaged with the different antigens. Activity of such molecules could vary between individuals (for example through genetic variation) linking the variation in the responses at the population level with the observed correlations.

However, the correlations we describe are not complete and response to one antigen did not predict the response to a different cytokines with 100% certainty. Thus response to one vaccine will not necessarily predict response to another.

We did not observe strong correlation between any of the three cytokines measured for any of the mycobacterial antigens or for PHA (although weak correlation was observed between IFN-γ and IL-13 responses to PHA and KMTB). This is perhaps not surprising as different subsets of T cell involved in antimycobacterial immunity produce different cytokines. At the level of a single cell, whilst some regulatory mechanisms will influence all cytokine functions, there are also specific factors for individual cytokines. Further studies of vaccinated infants are required to tease out the relationships between the different cytokine responses, including the study of single cell cytokine production in response to mycobacterial antigens.

A further limitation of this study in terms of clinical relevance is the single time point we studied. Further longitudinal follow-up data would be of key importance to assess whether the observed immune responses are sustained and whether the variability is sustained or has an impact on the kinetics of the immune response over time.

In summary, we have studied cytokine responses to neonatal BCG vaccination in a relatively large cohort comprising 236 infants. We conclude that cytokine responses to mycobacterial antigens in BCG vaccinated infants are heterogeneous. Of particular interest are the IFN-γ responses since this is an important marker of protective immunity used for testing new vaccines for TB. Many variables contribute to the observed findings. It is likely that host genetic factors underlie the observed variation in this population [19] and identification of specific genetic variants involved could lead to more strategic vaccination programmes and prevention for at risk individuals. However, in other settings the relative contributions of other non-genetic factors such as concurrent helminth infection and higher *M. tuberculosis* exposure may be higher. Further research is different populations is required.
